# Analyzing Temperature Distribution Patterns on the Facing and Backside Surface: Investigating Combustion Performance of Flame-Retardant Particle Boards Using Aluminum Hypophosphite, Intumescent, and Magnesium Hydroxide Flame Retardants

**DOI:** 10.3390/polym15234479

**Published:** 2023-11-21

**Authors:** Fangya Pan, Hongyu Jia, Yuxiang Huang, Zhilin Chen, Shanqing Liang, Peng Jiang

**Affiliations:** 1 Research Institute of Wood Industry, Chinese Academy of Forestry, Haidian District, Beijing 100091, China; panfangya@aliyun.com (F.P.); jiahongyu9417@163.com (H.J.); yxhuang@caf.ac.cn (Y.H.); chenzhilin@caf.ac.cn (Z.C.); liangsq@caf.ac.cn (S.L.); 2 Co-Innovation Center of Efficient Processing and Utilization of Forest Resources, Nanjing Forestry University, Nanjing 210037, China

**Keywords:** particle board, combustion performance, temperature distribution, flame-retardant mechanism

## Abstract

Particle boards are manufactured through a hot pressing process using wood materials (natural polymer materials) and adhesive, which find common usage in indoor decorative finishing materials. Flame-retardant particleboard, crucial for fire safety in such applications, undergoes performance analysis that includes assessing temperature distribution across its facing surface and temperature increase on the backside surface during facade combustion, yielding critical insights into fire scenario development. In this study, a compact flame spread apparatus is utilized to examine the flame retardancy and combustion behavior of particle boards, with a specific emphasis on the application of cost-effective flame retardants, encompassing aluminum hypophosphite (ALHP), an intumescent flame retardant (IFR) comprising ammonium polyphosphate (APP), melamine (MEL), and Dipentaerythritol (DPE), alongside magnesium hydroxide (MDH), and their associated combustion characteristics. The D_300°C_ values, representing the vertical distance from the ignition point (IP) to P_300°C_ (the temperature point at 300 °C farthest from IP), are measured using a compact temperature distribution measurement platform. For MDH/PB, APP + MEL + DPE/PB, and ALHP/PB samples, the respective D_300°C_ values of 145.79 mm, 117.81 mm, and 118.57 mm indicate reductions of 11.11%, 28.17%, and 27.71%, compared to the untreated sample’s value of 164.02 mm. The particle boards treated with ALHP, IFR, and MDH demonstrated distinct flame-retardant mechanisms. MDH/PB relied on the thermal decomposition of MDH to produce MgO and H_2_O for flame retardancy, while APP + MEL + DPE/PB achieved flame retardancy through a cross-linked structure with char expansion, polyphosphate, and pyrophosphate during combustion. On the other hand, ALHP/PB attained flame retardancy by reacting with wood materials and adhesives, forming a stable condensed P-N-C structure. This study serves as a performance reference for the production of cost-effective flame-resistant particleboards and offers a practical method for assessing its fire-resistant properties when used as a decorative finishing material on facades in real fire situations.

## 1. Introduction

Particle board (PB) is a type of engineered wood or non-wood material made from natural polymer materials with wood shavings as its primary raw material. It can be manufactured with or without the use of adhesive by a process involving paving, pre-pressing, and hot pressing to form sturdy panels. The most commonly used particle board typically consists of three layers: two surface layers made of fine wood shavings and a core layer made of coarser shavings. Urea-formaldehyde resin is the prevalent adhesive used in the production of particle board [1]. As a product of resourceful utilization, particle board exhibits remarkable qualities, including high strength, resistance to deformation, a flat surface, and stable physical and mechanical properties. This makes it a versatile material widely employed in furniture manufacturing, construction, packaging, and interior decoration of vehicles and ships, as well as in the production of household appliances, playing a crucial role in addressing the serious shortage of wood resources. In Austria (Kronospan), Canada (Norbord), China (Wanhua), and various other countries worldwide, the particle board industry has experienced rapid growth, with production steadily increasing year after year. Taking China as a prime example, since 2015, the particle board industry has garnered unprecedented attention and experienced substantial growth, primarily fueled by the booming custom furniture sector, indicating a promising developmental trajectory [2]. By the end of 2020, China boasted 348 particle board production lines, capable of producing an annual volume of 36.91 million cubic meters, contributing to the global annual production capacity of particle board, which reached 114.3 million m^3^.

In recent years, there has been a surge in fires caused by highly flammable interior decoration materials, significantly affecting people’s livelihoods and posing substantial risks to their personal safety and property. To address this issue, relevant laws and regulations impose higher requirements on the combustion performance grade of decoration materials used in various parts of most buildings and locations. Particle board, being both inflammable and flame-retardant, plays a critical role in fire prevention efforts. Initiating flame retardant measures from the source material and applying them during the production of particle board hold significant importance in preventing fires and slowing down their spread at various building sites. By incorporating flame-retardant particle board, we can effectively enhance fire safety and reduce the rapid propagation of fires.

Currently, research on flame-retardant particle board primarily focuses on combustion theory [3], flame retardant mechanisms [4,5], flame retardant agents [6,7,8,9,10], flame retardant treatment processes [4,11,12], and flame retardant property detection methods, among others. Previous studies have explored various types of flame retardants for particle board, including boron-based [7], metal-based [13], phosphonitryl [6], inorganic minerals [12], biomass flame retardants, intumescent flame retardants [8], nano flame retardants [14], and so on. Metal-based flame retardants are widely used in flame-retarding wood materials due to their cost-effectiveness. [15,16,17,18]. In particular, a flame-retardant particle board was developed using dried oil palm as the raw material, incorporating aluminum hydroxide and magnesium hydroxide (MDH) as flame retardants, achieving limiting oxygen indices of 28.55% and 27.95%, respectively [19]. The application of intumescent flame retardants was initially adopted for plastic products and later extended to wood materials, yielding improved flame retardancy results. Notably, ammonium polyphosphate (APP) intumescent flame retardants have shown excellent performance when applied to wood-based panels, exhibiting low heat release rates, minimal total heat release, limited smoke emission, a high limiting oxygen index, and overall superior flame retardancy properties [20]. Furthermore, aluminum hypophosphite (ALHP) is frequently employed as a plastic flame retardant [21,22,23,24,25], and when adding 10 wt% ALHP to polylactic acid (PLA) in the UL-94 test, it can achieve a V0 rating [26]. As for testing methods, several approaches are available to assess the flame-retardant performance of particle board, including ignitability, smoke generation, flame propagation, thermal analysis, and more [27,28,29]. Nevertheless, there remains a requirement for testing methodologies tailored to accurately discern the surface temperature distribution and the corresponding rise in rear temperature for flame-retardant particle board. Therefore, conducting research on the flame-retardant effects and mechanisms of particle board treated with different flame retardants and exploring novel flame-retardant detection methods holds great significance.

The combustion reactions of various parts within artificial boards differ, primarily categorized into the surface gas phase area, and materials progressing from the surface to the interior undergo carbonization, degradation, dehydration, and heating processes, respectively [30]. Thus, this research focuses on enhancing the flame resistance of particle boards by incorporating various flame retardants, achieved by considering the combustion behavior of artificial board materials and the properties of flame retardants in the preparation of a series of flame-retardant particle boards. In this study, samples of flame-retardant particle boards containing ALHP, IFR, and MDH have been prepared, and a combination of a compact temperature distribution measurement platform and a cone calorimeter was employed to evaluate the combustion behavior of flame-retardant particle boards, such as temperature distribution distance, temporal back temperature variations, heat release rate (HRR), and smoke production rate (SPR). Furthermore, a flame-retardant mechanism in the condensed phase was proposed, drawing upon the analysis of char residues following combustion and utilizing scanning electron microscopy (SEM), Raman spectrometry, and X-ray photoelectron spectroscopy (XPS). This study is geared towards offering valuable insights into the cost-effective flame-retardant mechanisms displayed by various flame-retardant particle boards, introducing novel flame-retardant testing approaches, and supplying theoretical backing for the practical implementation of flame retardants in wood-based materials, particularly in real fire scenarios.

## 2. Materials and Methods

### 2.1. Materials

In this experiment, wood shavings with a moisture content of 2–3% (measured in percentage) are utilized as the primary raw material. Urea-formaldehyde resin adhesive (UF), along with aluminum hypophosphate (Shandong Taixing New Material Co., Ltd., Jinan, China), DPE (Shanghai Aladdin Biochemical Technology Co., Ltd., Shanghai, China), APP, MEL and MDH (both from Shandong Changsheng flame retardant new material Co., Ltd., Dezhou, China), is incorporated into the experimental setup. The wood shavings and urea-formaldehyde resin adhesive are sourced from Shandong Xingang Enterprise Group Co., Ltd. (Linyi, China) Table 1 provides essential details regarding the adhesives employed in this study.

### 2.2. Manufacture of Particle Board

**Figure 1:**
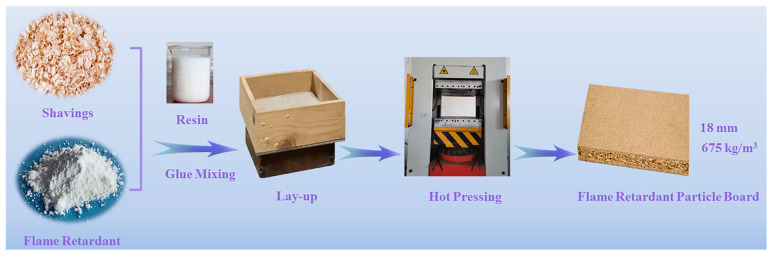
Schematic diagram of particle board manufacturing.

In this experiment, ordinary untreated and flame-retardant particle boards with a thickness of 18 cm and a density of 675 kg/m^3^ are prepared. The manufacturing process involved weighing, glue mixing, lay-up, and hot pressing, as shown in Figure 1. The shavings consisted of 34% on the surface and 66% in the core. The resin content is 10 wt% on the surface and 10.5 wt% in the core. The compositions of the untreated and flame-retardant particle boards are listed in Table 2. The hot pressing process included prepressing at 3 MPa and room temperature for 20 s, followed by hot pressing at 175 °C and 2.5 MPa for 8 min. The flame retardant is added in the same proportion to both the surface and core layers of the flame-retardant particle boards. The formulations for the untreated and flame-retardant particle boards are listed in Table 3.

### 2.3. Characterization

Limiting oxygen index (LOI): An oxygen index tester (JF-3, Jiangning Analysis Instrument Company, Nanjing, China) is used for measurement. The specimen size is 120 mm × 10 mm × thickness according to ASTM D2863-17 [31].

Combustion performance: The combustion behavior of the sample is evaluated using a cone calorimeter (Fire Testing Technology Ltd., East Grinstead, UK) in accordance with the ASTM E1354-17 standard [32]. The sample size is 100 mm × 100 mm × thickness. To conduct the experiment, the samples are positioned horizontally and subjected to a heat flux of 50 kW/m^2^. They are carefully wrapped in aluminum foil, leaving the upper surface exposed to the heater. The wrapped samples are then placed on a ceramic backing board, maintaining a distance of 35 mm from the cone base.

**Figure 2:**
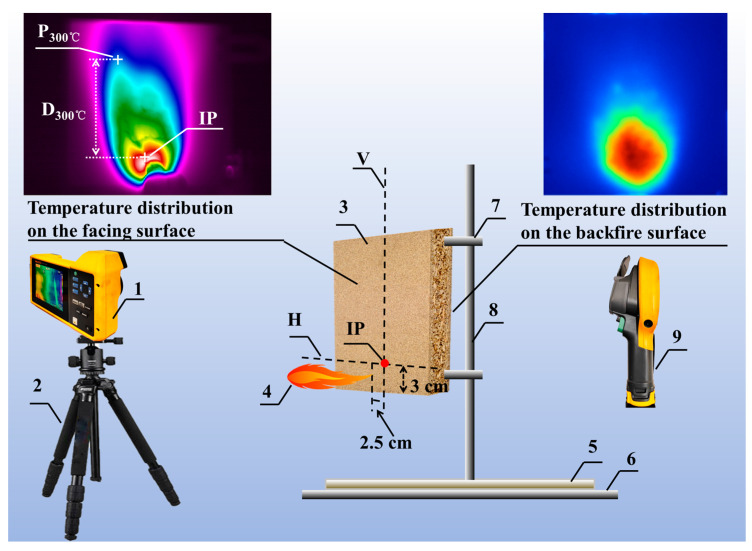
Schematic diagram of a small-scale temperature distribution measurement platform. 1—infrared thermal imager, 2—tripod, 3—sample, 4—spray gun, 5—gypsum board, 6—support pedestal, 7—fixture, 8—support, 9—hand-held temperature detector.

The distribution of the temperature: The experiment is conducted with a compact temperature distribution measurement platform, as shown in Figure 2, with the sample positioned perpendicular to the horizontal plane. The ignition point (IP) is defined as the intersection of the horizontal axis line (H), situated 3 cm above the lower edge of the sample facing the heat source, and the vertical axis line (V), passing through the center point of the material (at a point 3 cm above the midpoint of the lower edge of the sample facing the heat source).

The particle board was continuously lit, and a spray gun was employed, positioned 2.5 cm away from the ignition point. The resulting flame had a length of 10 cm. During the experiment, an infrared thermal imager (TiX580, Fluke, Shanghai, China) is employed to capture video footage of the facing surface, while a handheld temperature detector (Ti100, Fluke, Shanghai, China) is utilized to photograph the backside surface, with the experiment concluding when the maximum temperature of the backside surface reached 275 °C. The sample size in the experiment is 20 cm × 20 cm × 1.8 cm, and to prevent fire hazards arising from the high temperatures during combustion, a piece of gypsum board is placed underneath the support. Smart View 4.4 software is used to obtain the 300 °C temperature point farthest from IP (P_300°C_) on the facing surface of particle board and the highest temperature point on the backside surface. The vertical distance from P_300°C_ to IP (D_300°C_) is calculated, and Origin 8.5 software is employed for data analysis to obtain information such as D_300°C_ and the temporal evolution of backside temperature.

Physical and mechanical performance: The properties of particle board samples, including thickness, density, internal bonding strength, surface bonding strength, modulus of rupture (MOR), and modulus of elasticity (MOE), are determined following the guidelines outlined in the GB/T 17657-2013 standard [33] titled “Test methods of evaluating the properties of wood-based panels and surface-decorated wood-based panels.” This standard provides specific procedures and protocols for measuring and evaluating these characteristics of particle boards.

Morphology of char residue: The char residue morphology of untreated and flame-retardant particle boards is captured by a scanning electron microscope (Gemini SEM 300, ZEISS, Oberkochen, Germany) equipped with an energy-dispersive X-ray (EDX) sensor. Scanning electron microscope (SEM) samples are sputtered with gold using a SCD005 Sputter Coater from BAL-TEC(Switzerland) at a current of 40 mA for 180 s.

Raman spectrum of char residue: A Raman spectrometer (LabRAM HR Evolution, HORIBA, Paris, France) is used to study the graphitization degree of char residue. The excitation light source is 532 nm, and the scanning area is 50–4000 cm^−1^.

X-ray photoelectron spectroscopy (XPS) analysis of char residue: XPS analysis of char residue is conducted using a Thermo ESCALAB 250XI (ThermoFischer, Waltham, MA, USA), with an analysis chamber vacuum level of 4 × 10^−9^ mbar, an Al Kα ray excitation source (hv = 1486.6 eV), a working voltage of 14.6 kV, a filament current of 13.5 mA, and signal accumulation over 20 cycles. The test pass-energy is 20 eV, the step size is 0.1 eV, and the charge correction is performed with C1s = 284.8 eV as the combined energy standard.

## 3. Results

### 3.1. LOI Analysis

The LOI values for both untreated and flame-retardant particle boards have been listed in Table 4. The LOI value for untreated particle board stands at 26.4, indicating a heightened potential for fire hazards. However, when flame retardants ALHP, IFR, and MDH are introduced individually at a 10% mass fraction, LOI values experience significant increases, reaching 35.8, 33.7, and 29.4, respectively. These improvements mark substantial enhancements of 35.61%, 27.65%, and 11.36% in LOI values relative to untreated particle boards. These findings underscore the significant elevation in flame retardancy achieved through the application of ALHP, IFR, and MDH treatments. Table 4 further demonstrates that the most noteworthy LOI values, specifically 35.8 and 33.7, are attained with the inclusion of phosphorus-based ALPH and IFR flame retardants. Significantly, particle board inherently contains oxygen elements present in both the wood and urea-formaldehyde resin adhesive, with the observed enhancement in flame retardancy attributed to their interaction with phosphorus during combustion. Subsequent sections of this study will provide an in-depth exploration of the underlying flame-retardant mechanisms.

### 3.2. Temperature Distribution on the Facing Surface and the Temperature Rise on the Backside Surface

**Figure 3:**
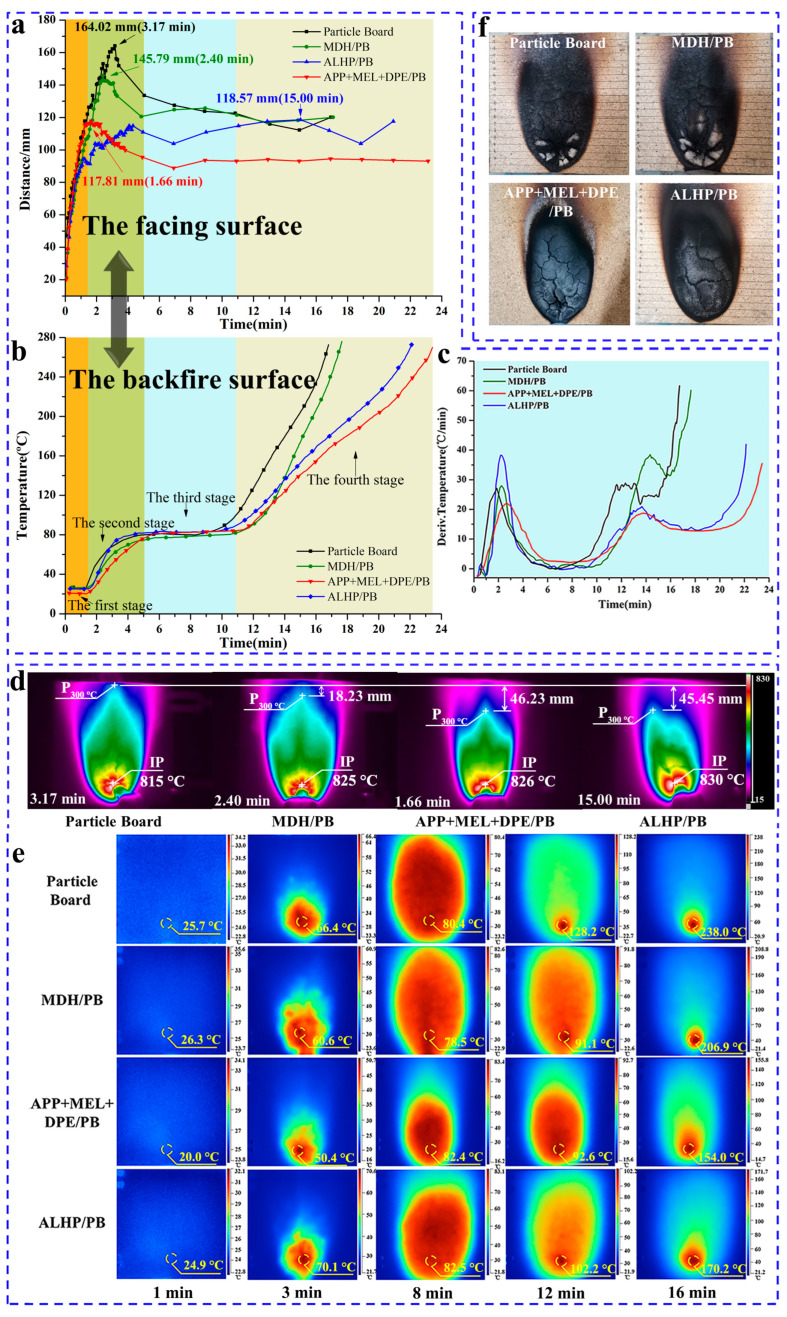
D_300°C_—Time curve (**a**); backside surface temperature rise curve (**b**); backside surface temperature rise rate curve (**c**); infrared images of the fire-facing surface (**d**) and backside surface (**e**) of untreated and flame-retardant particle boards; and digital images of char residues (**f**).

The temperature distribution on the fire-facing surface and the temperature rise on the backside of untreated and flame-retardant particle boards are investigated using a small-scale temperature distribution experimental platform. Figure 3a illustrates the temporal evolution of the vertical distance from P_300°C_ to IP (D_300°C_) for both untreated and flame-retardant particle boards, showing a rapid increase in D_300°C_ after ignition followed by stabilization within distinct ranges, attributed to heat propagation from the ignition source and thermal carbonization of the samples. For untreated particle board, the maximum D_300°C_ was achieved at approximately 164.02 mm (3.17 min after ignition), while the maximum D_300°C_ values and ignition times for MDH/PB, APP + MEL + DPE/PB, and ALHP/PB samples are 145.79 mm (2.40 min), 117.81 mm (1.66 min), and 118.57 mm (15.00 min), respectively. Significantly, the APP + MEL + DPE/PB samples displayed both the lowest peak D_300°C_ and a narrower stable region compared to the other samples, which can be attributed to the effective flame propagation and heat transfer inhibition of the IFR flame retardant. While the peak D_300°C_ of ALHP/PB samples ranked second only to that of IFR-treated samples, it consistently maintained a relatively high level during continuous ignition, indicating that ALHP is less effective than IFR flame retardants in suppressing flame propagation during particle board combustion and highlighting the distinct flame-retardant mechanisms of IFR and ALHP when applied to particle board.

Figure 3d displays infrared images depicting the maximum D_300°C_ of the untreated and flame-retardant particle boards. The images indicate the position of the 300 °C temperature point farthest from the IP (P_300°C_) and ignition point (IP), with the 300 °C region represented by a light blue color. Comparing the untreated particle board with MDH/PB, APP + MEL + DPE/PB, and ALHP/PB, the flame-retardant variants demonstrate a reduction in the maximum D_300°C_. Specifically, MDH/PB, APP + MEL + DPE/PB, and ALHP/PB samples reduce the distance by 18.23 mm (11.11% reduction), 46.23 mm (28.17% reduction), and 45.45 mm (27.71% reduction), respectively. The digital photos in Figure 3f depict the char residue following the combustion of the samples. In the cases of untreated particle board and MDH/PB samples, notable ash accumulation is evident at the ignition point (IP), and numerous cracks make the char easily detachable. In contrast, the char residues from the APP + MEL + DPE/PB sample, featuring minimal ash content and narrower cracks compared to other variants, effectively restrained flame spread on the approaching side and minimized heat transfer on the backfiring side, aligning with the trends observed in Section 3.3’s backside temperature rise curve. For ALHP/PB samples, wider combustion residue cracks are observed, leading to a delay of up to 300 °C.

The temperature permeability of flame-retardant particle boards in the direction of board thickness is evaluated by monitoring the temperature increase on the backside surface, with the experiment concluding upon reaching a temperature of 275 °C. Figure 3b,c depict the temperature rise and temperature rise rate, respectively, of untreated and flame-retardant particle boards under continuous ignition on the facing surface. According to Figure 3b, the untreated particle board reaches a backside surface temperature of 275 °C in approximately 16.80 min, while MDH/PB, APP + MEL + DPE/PB, and ALHP/PB attain the same temperature levels at 17.65 min, 23.43 min, and 22.13 min, respectively. IFR flame-retardant particle board samples effectively extend the time to reach 275 °C on the backside surface by 6.63 min, a 39.46% increase, which is crucial for fire emergency response.

Figure 3b illustrates four primary stages in the temperature rise over time on the backside surface, with each stage corresponding to distinct characteristics and behaviors that coincide with the rapid increase of D_300°C_ on the fire-facing surface. The second stage occurs near the peak of D_300°C_ on the fire-facing surface. The third stage signifies a relatively stable D_300°C_ on the fire-facing surface, while the fourth stage represents the primary phase of heat penetration into the material. Data regarding the duration of each stage and temperature rise rates, which were obtained from Figure 3c for both untreated and flame-retardant particle board samples, are listed in Table 5. The APP + MEL + DPE/PB sample exhibited the lowest temperature rise rate during the temperature rise on the backside fire surface, primarily in the second and fourth stages. Both the APP + MEL + DPE/PB and ALHP/PB samples exhibit longer backside surface temperature times, primarily due to extended durations in the third stage and the fourth stage compared with the MDH/PB sample. Figure 3e presents infrared images of the backside surface at various characteristic points during the four stages of untreated and flame-retardant particle boards. Examining the time-temperature thermal imaging maps corresponding to the second (3rd minute), third (8th minute), and fourth (12th and 16th minute) stages, it can be observed that there are differences in temperature and thermal imaging range of untreated and flame-retardant particle boards, with the APP + MEL + DPE/PB sample having the lowest temperature and the smallest high-temperature heat distribution range.

### 3.3. Cone Calorimeter

**Figure 4:**
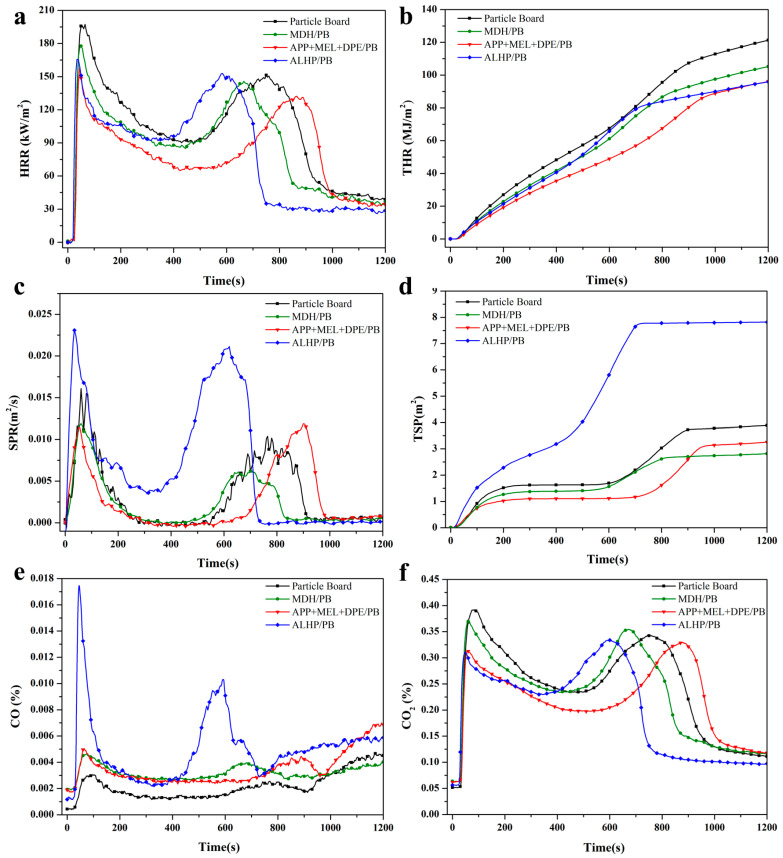
Heat release rates (**a**), total heat residual weight (**b**), smoke production rate (**c**), and total smoke production (**d**), CO concentration (**e**), and CO_2_ concentration (**f**) curves for untreated and flame-retardant particle boards.

To more explicitly demonstrate the fire risk and flammability of the particle board samples, cone calorimetry analysis (CONE) was employed. The heat release rate (HRR) and total release (THR) curves of the samples as a function of time are depicted in Figure 4a,b, and the key data received from CONE are listed in Table 6. The flame-retardant particle boards exhibit differences in ignition time (t_ign_, 21 s) and the time of the second peak heat release rate (t_pHRR2_, 750 s) compared to the untreated particle board. Specifically, APP + MEL + DPE/PB samples show an extension to 24 s and 865 s for t_ign_ and t_pHRR2_, respectively, corresponding to delay rates of 14.29% and 15.33%, respectively. APP + MEL + DPE/PB samples exhibit the most significant reduction in peak heat release rate (pHRR) and total heat release at 800 s (THR_800s_), with percentage decreases of 19.33% and 29.29% compared with the untreated ones, respectively. It is noteworthy that the second peak in this sample is also delayed, which is mainly due to IFR promoting the char formation of particle board materials. APP + MEL + DPE/PB samples exhibit the highest fire performance index (FPI) at 0.151 m^2^·s/kW, surpassing the untreated particle board by 41.67%, signifying that APP + MEL + DPE/PB samples present the lowest fire risk among the three types of flame-retardant particle boards.

Furthermore, Figure 4c,d present the smoke production rate (SPR) and total smoke production (TSP) curves. From the figure, it is evident that the peak smoke production rate (pSPR) and TSP values of MDH/PB are the lowest, measuring 0.012 m^2^/s and 2.82 m^2^, respectively, suggesting that magnesium hydroxide is more effective in suppressing smoke during wood combustion. In contrast, ALHP/PB samples exhibit notably higher pSPR and TSP values, measuring 0.025 m^2^/s and 7.82 m^2^, respectively, which represent approximately 2.08 times and 2.77 times the values observed in the untreated particle board. The variations in smoke production rate (SPR) and total smoke production (TSP) among flame-retardant particle board samples can be attributed to the release of CO and CO_2_, a correlation supported by the synchronization of peak CO and CO_2_ concentrations with the peak SPR, as depicted in Figure 4e,f. Simultaneously, it is evident that the ALHP/PB samples display the highest pSPR and TSP values, which can be attributed to the emission of CO.

### 3.4. Physical and Mechanical Performance

The physical and mechanical characteristics of untreated particle board and flame-retardant particle board are detailed in Table 7. It is evident that the incorporation of flame retardants has led to varying degrees of reduction in the physical and mechanical properties of the particle boards. The lower internal and surface bonding strengths of ALHP/PB samples, in comparison to the other two flame-retardant particle boards, may be attributed to the acidic nature of aluminum hypophosphate (pH 3–5), which affects the crosslinking degree of the urea-formaldehyde resin dimer and, in turn, impacts bonding strength. Additionally, ALHP exhibits poor compatibility with the substrate, infiltrating the bonding interface between the adhesive and the unit shavings, thereby diminishing the bonding points. In contrast, the APP + MEL + DPE/PB samples show the highest MOR and MOE due to melamine’s presence in the extended flame-retardant system, promoting cross-linking reactions and leading to a stronger three-dimensional network structure, thus enhancing cohesion, rigidity, and resin bonding strength, ultimately boosting the MOR and MOE of the sheet [34]. Remarkably, the considerably higher absorption thickness expansion rate in MDH/PB samples, in comparison to the other two flame-retardant particle boards, may be attributed to the inherent hydrophilic nature of the hydroxyl groups present in magnesium hydroxide.

### 3.5. Mechanism Analysis

#### 3.5.1. Morphology of Char Residue

**Figure 5:**
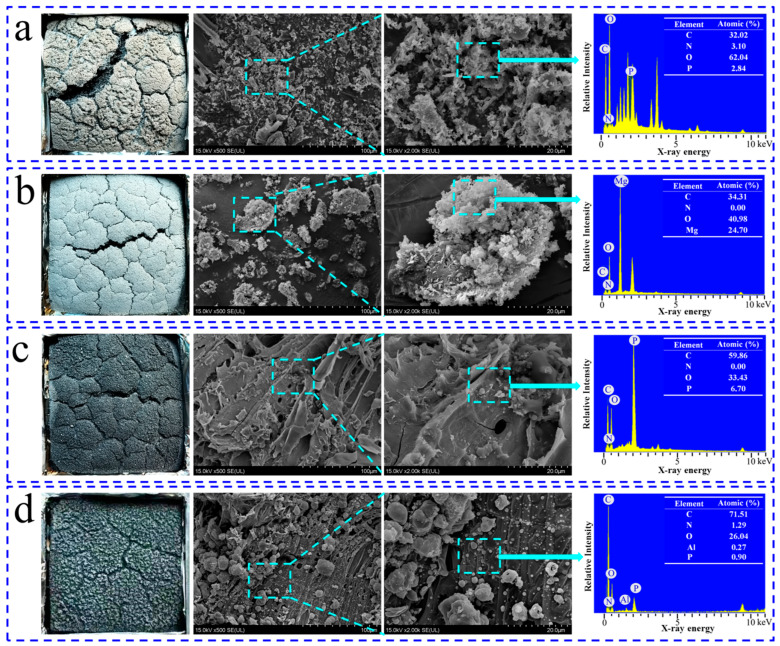
Digital images, SEM images, and EDX spectra of char residues of untreated particle board (**a**), MDH/PB (**b**), APP + MEL + DPE/PB (**c**), and ALHP/PB (**d**).

Based on the above analysis, it is interesting to explore the difference in the char residuals between untreated and flame-retardant particle boards. Figure 5 illustrates the digital images, SEM images, and EDX spectra of the combustion residues of untreated and flame-retardant particle boards. As depicted in Figure 5a, the char layer of untreated particle board appears gray after complete wood material combustion, signifying weak char layer strength, with SEM images additionally revealing a loose and collapsed wood fiber microstructure post-combustion. In contrast, the char layer in the MDH/PB samples exhibited a noticeably lighter gray shade, primarily attributed to the lingering magnesium oxide post-combustion and dehydration of MDH. Nonetheless, the char layer structure of the MDH/PB samples is denser compared to the untreated particle board. Following ALHP treatment, the residual char color on the flame-retardant particle boards turned black, with a slight presence of white material on the surface. In contrast to MDH treatment, there is a notable enhancement in the density of the char layer, and the SEM images distinctly displayed the presence of granular material. Concerning the IFR-treated samples, Figure 5c demonstrates a thicker char layer structure, signifying enhanced strength, with SEM images highlighting a significant improvement in densification. According to EDX spectrum analysis, particle boards treated with various flame retardants (MDH, IFR, and ALHP) exhibited varying levels of characteristic elements (Mg, Al, and P/Al elements) remaining after combustion. It is worth emphasizing that the relative atomic percentages of Al and P in the ALHP/PB samples are relatively low, primarily due to the release of P in the form of PH_3_ flue gas, thereby enhancing its flame-retardant properties in the gas phase [24]. This finding is consistent with the relatively high pSPR values observed in the ALHP/PB samples (Figure 4c).

#### 3.5.2. Raman Spectrum of Char Residue

**Figure 6:**
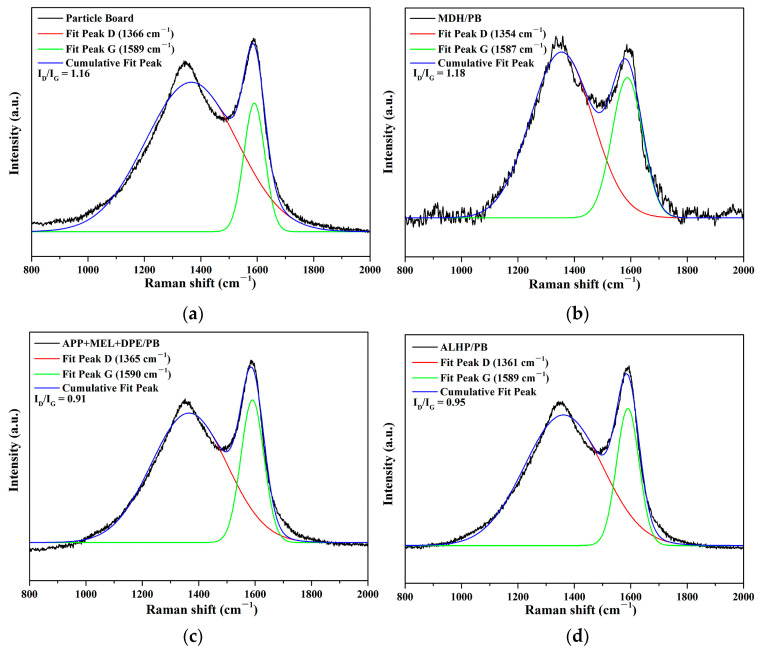
Raman spectra of char residues of untreated particle board (**a**), MDH/PB (**b**), APP + MEL + DPE/PB (**c**), and ALHP/PB (**d**).

Raman spectroscopy is a widely employed technique to assess the graphitization degree of carbon materials. In the spectra, the D band corresponds to the vibration of disordered graphite’s sp^3^ hybrid carbon atoms, while the G band represents the vibration of sp^2^ hybrid carbon atoms in a 2D hexagonal lattice [35]. The ID/IG ratio serves as an indicator for evaluating the quality of carbon materials [36]. A lower ID/IG value indicates a higher degree of graphitization and fewer defects. Figure 6 displays the Raman spectra of the combustion residues from untreated and flame-retardant particle boards. The ID/IG values for untreated particle board, MDH/PB, APP + MEL + DPE/PB, and ALHP/PB samples are as follows: 1.16, 1.18, 0.91, and 0.95, respectively. Notably, APP + MEL + DPE/PB exhibit the lowest ID/IG value, signifying a higher degree of graphitization and improved char residue quality compared to ALHP/PB. Additionally, the ID/IG value of MDH/PB is similar to that of the untreated sample, indicating that MDH does not contribute to enhancing the quality of char residue.

#### 3.5.3. XPS Analysis of Char Residue

**Figure 7:**
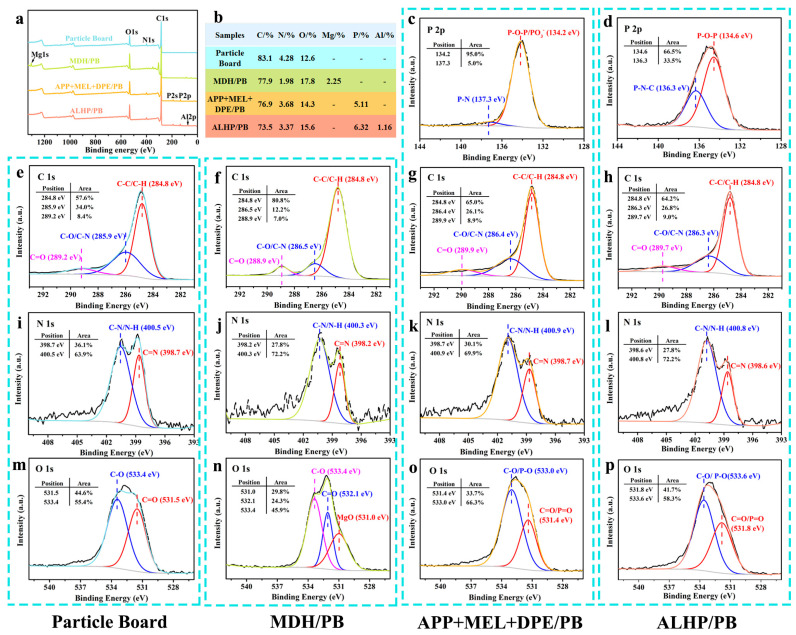
XPS survey spectra, X-ray photoelectron spectroscopy date, and high-resolution XPS spectra of the char residues produced by untreated particle board (**a**,**b**,**e**,**i**,**m**), MDH/PB (**a**,**b**,**f**,**j**,**n**), APP + MEL + DPE/PB (**a**–**c**,**g**,**k**,**o**), and ALHP/PB (**a**,**b**,**d**,**h**,**l**,**p**) after the CONE.

X-ray photoelectron spectroscopy (XPS) tests are conducted to analyze the chemical structure and composition of the char layer formed by flame-retardant particle boards, and the related results are exhibited in Figure 7. In the full survey XPS spectra of the char residue of untreated and flame-retardant particle boards, characteristic peaks attributed to Mg 1s (1304.9 eV), P 2s (192.25 eV), P 2p (136.31 eV), and Al 2p (76.18 eV) can be clearly observed.

As shown in Figure 7b, compared with the content of various elements of untreated and flame-retardant particle boards, the content of C and N in the Particle Board sample decreased and the content of O increased, suggesting that the addition of MDH, IFR, and ALHP played a role in protecting the matrix during combustion. The C 1s, N 1s, O 1s, and P 2p fine spectra of the char layer are fitted by peak fitting for Particle Board, MDH/PB, APP + MEL + DPE/PB, and ALHP/PB samples. The C 1s spectra of untreated and flame-retardant particle board samples exhibited three fitting peaks: 284.8 eV corresponding to C-C/C-H groups, 285.9–286.5 eV corresponding to C-O and C-N groups in the crosslinked structure, and 288.9–289.9 eV corresponding to completely oxidized C=O groups [37,38,39,40,41]. The peaks of the N 1s high resolution spectrum of untreated and flame-retardant particle board samples at 398.2–398.7 eV and 400.3–400.9 eV are assigned to C=N and pyrrole groups [42,43,44], respectively, and the content of C=N and pyrrole groups has changed, proving that the addition of flame retardant promotes the char formation of urea-formaldehyde resin adhesive in particle board during combustion. The peaks of the O 1s high resolution spectrum of MDH/PB samples at 532.1 eV, 533.4 eV, and 531.0 eV were assigned to C=O, C-O, and MgO groups, proving the decomposition of Mg(OH)_2_ into MgO during combustion [45,46,47]. C=O/P=O and C-O/P-O groups can be found in the O 1s high resolution spectrum of APP + MEL + DPE/PB (531.4 eV and 533.0 eV) and ALHP/PB (531.8 eV and 533.6 eV) samples [38,45]. The P 2p spectra of APP + MEL + DPE/PB and ALHP/PB char residue displayed two fitting peaks. The P 2p binding energy at 134.2 eV for APP + MEL + DPE/PB char residue is attributed to P-O-P/PO^3−^ in cross-linked polyphosphates and pyrophosphates [37,42,45], while 137.3 eV is attributed to P-N [48]. The P 2p binding energy for ALHP/PB char residue is attributed to P-O-P at 134.6 eV and P-N-C at 136.3 eV [49], indicating the formation of a relatively stable P-N-C structure due to the reaction of aluminum hypophosphate with shavings and adhesives.

**Figure 8:**
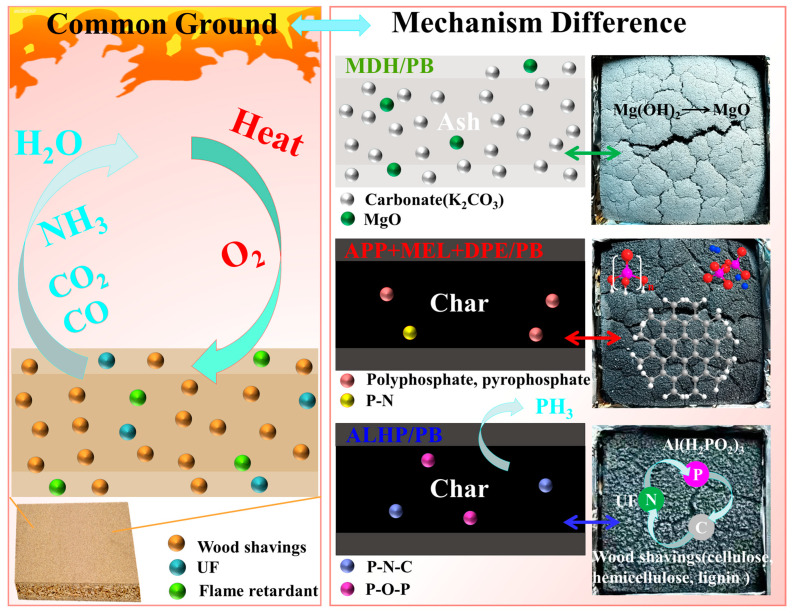
Schematic illustration of a flame-retardant mechanism for flame-retardant particle boards.

By analyzing the chemical composition and microstructure of char residue, the possible flame-retardant mechanism of three cost-effective particle boards has been elucidated (as depicted in Figure 8). Flame-retardant particle boards prepared using MDH, IFR, and ALHP all emit CO, CO_2_, H_2_O, and NH_3_ during combustion, exerting flame-retardant effects in the gas phase. Notably, these three flame-retardant particle boards demonstrated distinct combustion mechanisms. Mg(OH)_2_ exhibits limited reactivity with the substrate during combustion, leading to the formation of MgO, which persists in the ash. In contrast, the IFR compounds form a linked structure with wood and the urea-formaldehyde resin, resulting in the production of polyphosphates, pyrophosphates, phosphorus nitrogen compounds, etc., thereby enhancing flame-retardant performance. ALHP reacts with both shavings and urea-formaldehyde adhesives, establishing a relatively stable P-N-C structure, promoting char formation, and generating gaseous PH_3_.

## 4. Conclusions

In this study, we employed infrared imaging devices and other instruments to construct a small-scale temperature distribution experiment platform, enhancing the combustion performance testing methods for artificial board materials. IFR flame-retardant particle board has excellent ability to inhibit temperature diffusion and extend the time it takes for the backside temperature to rise, which is of great significance for emergency rescue work in the event of fire accidents. The self-made temperature distribution experiments revealed that APP + MEL + DPE/PB and ALHP/PB are more effective in reducing the maximum D_300°C_ and extending the back temperature time. Specifically, the maximum temperature diffusion distance is reduced by 46.23 mm (28.17% decrease) and 45.45 mm (27.71% decrease), while the back temperature time is extended by 6.63 min (39.46% extension) and 5.33 min (31.73% extension), respectively. APP + MEL + DPE/PB showed the best performance in reducing pHRR and THR_800_, with decreases of 19.33% and 29.29%, respectively. SEM, Raman spectra, and XPS analyses revealed that MDH/PBs flame retardant mainly depended on the thermal decomposition of Mg(OH)_2_ into MgO and H_2_O without char formation. In contrast, APP + MEL + DPE/PB expanded into char, forming a cross-linked structure with polyphosphate and pyrophosphate, while ALHP/PB reacted with particles and adhesive to form a stable condensed-phase P-N-C structure.

This study sheds light on flame-retardant mechanisms and combustion behaviors in various flame-resistant particleboards, providing insights for cost-effective production and practical evaluation as decorative building facade materials, especially in real fire scenarios. Moreover, this study’s results can guide manufacturers, architects, and builders in selecting appropriate flame-resistant particleboards for different construction projects, ensuring adherence to fire safety regulations, and promoting the development of safer and more fire-resistant structures in real-world scenarios.

## Figures and Tables

**Table 1:** Urea—formaldehyde resin adhesive basic information.

Samples	Solid Content (%)	Viscosity (Pa·s)	pH
Surface adhesive	59.2	39.2	7.7
Core layer adhesive	65.0	150.0	8.0

**Table 2:** The compositions of untreated and flame-retardant particle boards.

Samples	Surface Layer (34 wt%)			Core Layer (66 wt%)		
	Shavings (wt%)	Resin (wt%)	Flame Retardant (wt%)	Shavings (wt%)	Resin (wt%)	Flame Retardant (wt%)
Particle Board	100	10	-	100	10.5	-
MDH/PB	100	10	10	100	10.5	10
APP + MEL + DPE/PB	100	10	10	100	10.5	10
ALHP/PB	100	10	10	100	10.5	10

**Table 3:** Formulations of untreated and flame-retardant particle boards.

Samples	MDH (wt%)	Intumescent Flame Retardant (IFR) (wt%)			ALHP (wt%)
		APP (wt%)	MEL (wt%)	DPE (wt%)	
Particle Board	-	-	-	-	-
MDH/PB	10	-	-	-	-
APP + MEL + DPE/PB	-	5	2.5	2.5	
ALHP/PB	-	-	-	-	10

**Table 4:** Limit oxygen index of untreated and flame-retardant particle boards.

Samples	Particle Board	MDH/PB	APP + MEL + DPE/PB	ALHP/PB
LOI/%	26.4 ± 0.1 d	29.4 ± 0.2 c	33.7 ± 0.1 b	35.8 ± 0.3 a

Different letters followed the mean values, which means a significant difference at the 0.05 level (*p* < 0.05).

**Table 5:** Key data from the back temperature curve of untreated and flame-retardant particle boards.

Samples	The First Stage (min)	The Second Stage (min)	The Third Stage (min)	The Fourth Stage (min)	Temperature Rise Rate (°C/min)	
					The Second Stage	The Fourth Stage
Particle Board	1.30 ± 0.08 b	3.45 ± 0.23 b	3.57 ± 0.23 c	8.48 ± 0.59 b	27.11 ± 1.89 b	61.64 ± 5.32 a
MDH/PB	1.43 ± 0.09 ab	3.95 ± 0.25 a	5.02 ± 0.32 b	7.25 ± 0.50 b	27.98 ± 2.02 b	60.16 ± 4.21 a
APP + MEL + DPE/PB	1.47 ± 0.08 a	4.15 ± 0.23 a	5.73 ± 0.35 a	12.08 ± 0.80 a	21.94 ± 1.52 c	35.45 ± 2.38 b
ALHP/PB	1.55 ± 0.08 a	2.2 ± 0.13 c	6.25 ± 0.36 a	12.13 ± 0.85 a	38.27 ± 2.87 a	41.96 ± 2.94 b

Different letters followed the mean values, which means a significant difference at the 0.05 level (*p* < 0.05).

**Table 6:** Key data from the cone calorimeter test of untreated and flame-retardant particle boards.

Samples	t_ign_ (s)	t_pHRR1_ (s)	pHRR_1_ (kW/m^2^)	t_pHRR2_ (s)	pHRR_2_ (kW/m^2^)	THR_800_ (MJ/m^2^)	Residual Weight (%)	FPI (m^2^·s/kW)	pSPR (m^2^/s)	TSP (m^2^)
Particle Board	21 ± 2 a	65 ± 5 a	197.36 ± 13.06 a	750 ± 31 b	151.31 ± 9.87 a	95.48 ± 10.46 a	22.01 ± 1.54 b	0.106 ± 0.007 bc	0.016 ± 0.001 b	3.89 ± 0.26 b
MDH/PB	21 ± 2 a	50 ± 3 b	177.74 ± 11.74 ab	665 ± 29 c	145.83 ± 9.63 b	86.66 ± 9.42 a	28.19 ± 1.84 a	0.118 ± 0.007 b	0.012 ± 0.001 c	2.82 ± 0.18 c
APP + MEL + DPE/PB	24 ± 2 a	45 ± 3 b	159.21 ± 10.25 b	865 ± 35 a	132.32 ± 8.66 b	67.51 ± 7.24 b	29.59 ± 2.07 a	0.151 ± 0.009 a	0.012 ± 0.001 c	3.26 ± 0.23 c
ALHP/PB	16 ± 1 b	35 ± 3 c	165.74 ± 10.85 b	585 ± 24 d	152.88 ± 10.09 a	83.98 ± 9.15 ab	30.20 ± 2.11 a	0.097 ± 0.006 c	0.023 ± 0.002 a	7.82 ± 0.45 a

Different letters followed the mean values, which means a significant difference at the 0.05 level (*p* < 0.05).

**Table 7:** Physical and mechanical data of untreated and flame-retardant particle boards.

Samples	Thickness (mm)	Density (kg·m^3^)	Internal Bonding Strength (MPa)	Surface Bonding Strength (MPa)	MOR (MPa)	MOE (MPa)	Absorption Thickness Expansion Rate (%)
Particle Board	17.72 ± 1.95 a	669.83 ± 60.21 a	0.89 ± 0.12 a	1.10 ± 0.21 a	15.11 ± 1.43 a	2489.67 ± 290.56 a	13.54 ± 1.47 c
MDH/PB	17.77 ± 2.00 a	657.71 ± 58.13 a	0.57 ± 0.02 b	0.64 ± 0.08 bc	7.39 ± 0.73 b	1757.00 ± 167.14 b	62.22 ± 5.38 a
APP + MEL + DPE/PB	17.70 ± 2.12 a	659.71 ± 58.31 a	0.47 ± 0.19 b	0.67 ± 0.18 b	10.02 ± 3.31 b	1978.00 ± 401.40 bc	22.24 ± 3.24 b
ALHP/PB	17.69 ± 1.94 a	654.61 ± 59.86 a	0.21 ± 0.02 c	0.37 ± 0.10 c	7.47 ± 0.47 b	1342.00 ± 100.44 c	22.47 ± 1.74 b

Different letters followed the mean values, which means a significant difference at the 0.05 level (*p* < 0.05).

## Data Availability

All data included in this study are available upon request by contacting the corresponding author.
